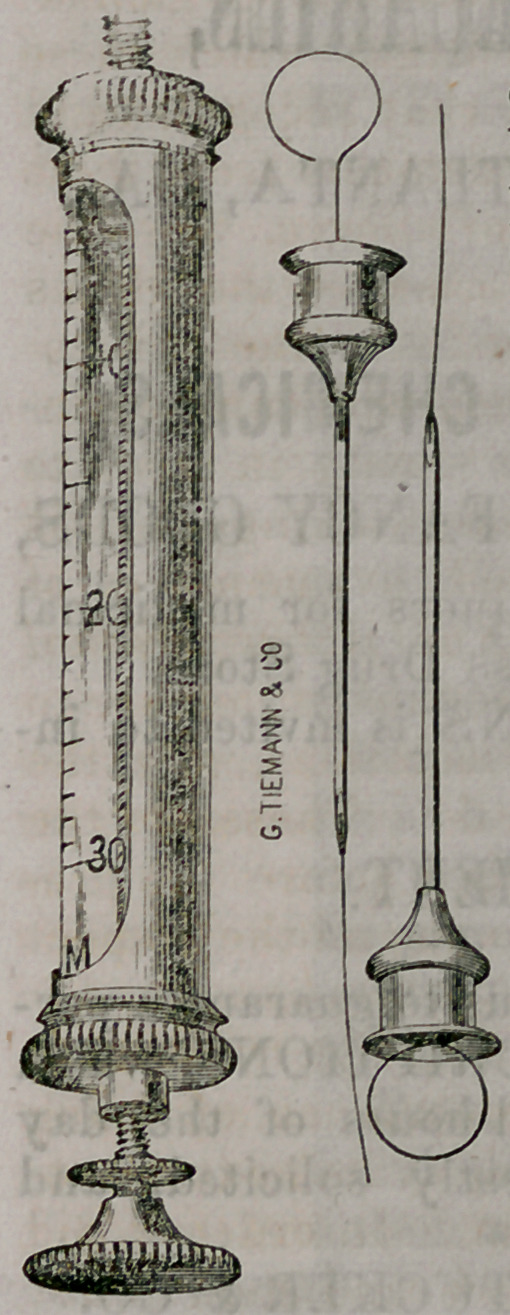# New Hypodermic Syringe

**Published:** 1871-05

**Authors:** 


### NEW HYPODERMIC SYRINGE.

We present our readers an electrotype view of a new Hypodermic Syringe, which is made by that excellent instrument-manufacturing house of Geo. Tiemann & Co., New York. The Mess. Tiemann have presented us with one of these instruments, for which we return them our sincere thanks. Their new Hypodermic Syringe consists of a graduated glass barrel, protected by a silver cylinder, frenestrated for the purpose of allowing the operator to see the graduated glass barrel, when using the instrument. The piston is arranged so as to fit in a tap by a screw its entire length, so that the fluid used for injection may be thrown gradually and regularly under the skin. It is a valuable instrument. No practitioner, in our judgement, should be without his Hypodermic Syringe, and we advise our friends to purchase the one now offered them by Geo. Tiemann & Co., as being the best in use. In this connectien, we beg to call the attention of our readers to the articles of our friend, Dr. W. A. Greene, of Americus, Ga. Dr. Greene has, doubtless, had a more general and extensive experience in the employment of hypodermic medication than any one gentleman in the country, and his contributions on this subject are highly interesting and satisfactory. We hope our friends will give the subject the attention it demands, and by all means, secure at once one of the excellent instruments of Tiemann & Co.

## Figures and Tables

**Figure f1:**